# An improvement in skeletal muscle mitochondrial capacity with short‐term aerobic training is associated with changes in Tribbles 1 expression

**DOI:** 10.14814/phy2.14416

**Published:** 2020-06-19

**Authors:** Rick B. Vega, Bram Brouwers, Stephanie A. Parsons, Natalie A. Stephens, Maria F. Pino, Andrew Hodges, Fanchao Yi, Gongxin Yu, Richard E. Pratley, Steven R. Smith, Lauren M. Sparks

**Affiliations:** ^1^ Translational Research Institute AdventHealth Orlando FL USA; ^2^ Sanford Burnham Prebys Medical Discovery Institute La Jolla CA USA

**Keywords:** aerobic exercise training, mitochondrial capacity, skeletal muscle, Tribbles 1

## Abstract

Exercise training and physical activity are known to be associated with high mitochondrial content and oxidative capacity in skeletal muscle. Metabolic diseases including obesity and insulin resistance are associated with low mitochondrial capacity in skeletal muscle. Certain transcriptional factors such as PGC‐1α are known to mediate the exercise response; however, the precise molecular mechanisms involved in the adaptation to exercise are not completely understood. We performed multiple measurements of mitochondrial capacity both in vivo and ex vivo in lean or overweight individuals before and after an 18‐day aerobic exercise training regimen. These results were compared to lean, active individuals. Aerobic training in these individuals resulted in a marked increase in mitochondrial oxidative respiratory capacity without an appreciable increase in mitochondrial content. These adaptations were associated with robust transcriptome changes. This work also identifies the Tribbles pseudokinase 1, *TRIB1*, as a potential mediator of the exercise response in human skeletal muscle.

## Graphical abstract

An 18‐day aerobic exercise training regimen increased skeletal muscle mitochondrial capacity and Tribbles 1 expression. These adaptations were associated with robust transcriptome changes. This work also identifies the Tribbles pseudokinase 1, TRIB1, as a potential mediator of the exercise response in human skeletal muscle.

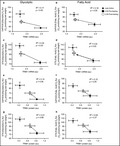

## 1 INTRODUCTION

Impairments in skeletal muscle (muscle) mitochondrial capacity adversely impact muscle function and whole‐body metabolic health. Associations between impaired muscle mitochondrial capacity and the development and presence of obesity and insulin resistance have been frequently described (Bajpeyi et al., 2011; Phielix et al., 2012; Sparks et al., 2005, 2013). Indeed, in comparison to healthy active individuals, those with sedentary behavior, obesity, and insulin resistance manifest lower skeletal muscle mitochondrial capacity (Bajpeyi et al., 2011; Meex et al., 2010; Phielix et al., 2008; Sparks et al., 2013). Impaired mitochondrial capacity can originate from derangements in intrinsic mitochondrial oxidative capacity (Phielix et al., 2008; Ritov et al., 2010) or from more quantitative deficits such as mitochondrial content and density (Bajpeyi et al., 2011; Kelley, He, Menshikova, & Ritov, 2002). Exercise training is an effective means to improve muscle mitochondrial capacity, glycemic control, and insulin sensitivity (Brouwers et al., 2018; Church et al., 2010; Meex et al., 2010; Sparks et al., 2013). It was recently described that people with type 2 diabetes require exercise training‐induced improvements in muscle mitochondrial capacity to obtain metabolic benefit from exercise training (Stephens et al., 2018). The exact molecular mechanisms that underlie differences in muscle mitochondrial capacity and are responsive to exercise, however, are not yet fully understood. Peroxisome proliferator‐activated receptor gamma coactivator 1‐alpha (PGC‐1α) is a key regulator of mitochondrial biogenesis and energy metabolism in skeletal muscle (Gan, Fu, Kelly, & Vega, 2018). Lower levels of PGC‐1α and muscle mitochondrial capacity are associated with obesity and diabetes in humans (Mootha et al., 2003; Patti et al., 2003). Likewise, increased PGC‐1α gene expression with exercise training has been suggested to play a key role in the exercise training‐induced improvements in muscle mitochondrial capacity (Fernandez‐Marcos & Auwerx, 2011; Lira, Benton, Yan, & Bonen, 2010). Some studies, however, have found exercise training‐induced improvements in muscle mitochondrial capacity before PGC‐1α gene expression was elevated (Wright et al., 2007). As such, additional molecular regulators that might play a role in the early adaptations of muscle mitochondria to exercise remain to be identified.

In the present study, we performed a comprehensive assessment of mitochondrial capacity using both in vivo and ex vivo gold‐standard techniques in Lean Active (LA) and Lean/Overweight Sedentary (LOS) individuals at baseline and in LOS following an aerobic interval exercise training program of 18 days. In parallel, we performed unbiased transcriptomic analyses in skeletal muscle to identify putative regulators of skeletal muscle mitochondrial capacity.

## 2 RESEARCH DESIGN AND METHODS

### 2.1 Participants

Sixteen participants completed the study, who were classified as Lean Active (LA, *n* = 7 males) and Lean/Overweight Sedentary (LOS, *n* = 9; *n* = 3 males). LOS individuals were not engaged in a regular exercise program. LA individuals had VO_2_max ≥45 ml kg min^−1^ and had to be engaged in a minimum of four cumulative hours of moderate to vigorous intensity aerobic exercise over no less than three days per week. Body weight was stable (<3 kg body weight change) during the last 8 weeks prior to enrollment. People with a history of type 2 diabetes and renal, cardiac, liver, lung, or neurological disease or any other medical condition that would interfere with clinical and laboratory measurements were excluded. Individuals were not allowed to take drugs or supplements that are known to affect energy metabolism or body weight and had to be able to adhere to the study protocol. The study protocol was approved by the AdventHealth Institutional Review Board and was carried out in accordance with the Declaration of Helsinki. All participants provided their written consent. This trial is registered at ClinicalTrials.gov, NCT01911091.

### 2.2 Study design

All participants underwent a VO_2_max test and anthropometric measurements, after which they were placed on a stabilization diet (35% fat, 55% carbohydrates, 15% protein) for two days with meals provided. Participants were presented to the clinical research unit at the Translational Research Institute (TRI; Orlando, FL) in the fasted state for Dual Energy X‐ray Absorptiometry (DEXA) scans and 31‐Phosphorus Magnetic Resonance Spectroscopy (^31^P‐MRS) for maximal ATP synthesis rate testing (ATPmax). Fasting blood samples were also collected. The following morning, a muscle biopsy was performed in the fasted state. Following baseline measurements, LOS individuals engaged in an exercise protocol for 18 days. Baseline measurements were repeated 3 days after the last exercise bout.

### 2.3 Exercise protocol

LOS individuals underwent a modified training protocol from a previously established exercise protocol during which participants exercised 18 of 21 consecutive days (Costford et al., 2010). Briefly, participants performed alternating day sessions of continuous and interval aerobic training. Continuous aerobic training was performed on a treadmill (T‐80, Vision Fitness) and increased each week by 30 min in duration from 30 to 90 min. Exercise intensity remained fixed at 70% of baseline VO_2_max over the course of the protocol. Interval training was performed on an upright cycling ergometer (Corival, Lode B.V.) and increased in target intensity by 10% each week from 60%–80% of VO_2_max. All exercise sessions were supervised on site at the TRI at AdventHealth.

### 2.4 Maximal mitochondrial capacity in vivo (ATPmax)

ATPmax was determined by measurement of the phosphocreatine (PCr) recovery rate in quadriceps muscle with ^31^P‐MRS using a 3T Philips Achieva magnet (Philips Healthcare), as previously described (Bajpeyi et al., 2011; Blei, Conley, & Kushmerick, 1993; Jubrias, Crowther, Shankland, Gronka, & Conley, 2003). Briefly, a ^31^P surface coil was used to measure PCr, ATP, and phosphorus (P_i_) using standard one pulse acquisition. ^31^P spectra were measured every 6–7.5 s for the duration of the experiment. While participants remained still, a fully relaxed ^31^P spectrum was obtained. The participant was then asked to kick against straps around the lower leg (i.e. contract the quadriceps muscle) for up to 45 s. Participants then remained still for an additional 5 min during PCr recovery. The PCr time constant (τ), and PCr level in oxygenated muscle at rest were used to measure ATPmax.

### 2.5 VO_2_max

VO_2_max was determined by a cycling ergometer exercise test (Lode B.V.). Participants were supervised and monitored according to the American College of Sports Medicine (ACSM) Guidelines for Exercise Testing and Prescription. As previously described (Costford et al., 2010), participants began exercising at moderate intensity and increased resistance until the point of exhaustion. The participant was considered to have reached maximal aerobic capacity when a plateau or decline in oxygen consumption was demonstrated. In addition, a respiratory exchange ratio (RER) ≥1.10 and an increase in heart rate to within 10 beats of the age‐predicted maximum, or a rate of perceived exhaustion (RPE) ≥17 on a scale of 6–20, also accompanied the achievement of maximal aerobic capacity.

### 2.6 Body composition

Body fat mass and lean body mass were measured by DEXA as previously described (Bajpeyi et al., 2011), using a GE Lunar iDXA whole‐body scanner (GE Lunar Healthcare) and analyzed with enCORE Windows‐based user interface.

### 2.7 Blood analyses

Fasted blood samples were obtained by venipuncture for laboratory analysis of plasma levels of glucose, insulin, HbA1c, high density lipoprotein (HDL), low density lipoprotein (LDL), very low density lipoprotein (VLDL), cholesterol, and triglycerides (Sparks et al., 2013).

### 2.8 Skeletal muscle tissue biopsy

Skeletal muscle biopsies were performed in a fasted state from the *vastus lateralis* as previously described (Bergstrom, 1975; Sparks et al., 2013). Tissue samples were snap frozen or processed ex vivo for ^14^C‐labeled substrate oxidation assays and O_2_ consumption measurements. Additional samples were embedded in Tissue‐Tek O.C.T. compound (Sakura Tissue‐Tek, Alphen a/d Rijn) and were frozen in liquid‐nitrogen cooled isopentane, stored at −80°C, and later used for fiber typing.

### 2.9 O_2_ consumption in muscle fibers

*Muscle fiber preparation*. Fresh muscle tissue (~20 mg) was placed in ice‐cold biopsy preservation solution (BIOPS; Oroboros instruments) immediately following retrieval from the biopsy. Muscle fibers were dissected and permeabilized in BIOPS buffer with saponin (5 mg/ml) for 30 min, then washed three times in mitochondrial respiration media (MiR05; OROBOROS instruments, Austria). All buffers were kept ice‐cold during processing of the muscle fibers. Permeabilized muscle fibers were blotted and measured for wet weight, then immediately transferred to the oxygraph‐2k (OROBOROS instruments, Austria) for measurement of respiration. 1.3–1.5 mg of fibers were placed in each chamber containing MiR05 at 37°C. O_2_ concentration in each chamber was maintained between 300 and 480 µM to prevent oxygen limitations of muscle fiber respiration.

### 2.10 Respiration protocol

Respiration was evaluated by titration of either glycolytic (2 mM malate +10 mM glutamate) or lipid (2 mM malate + 40 µM palmitoyl carnitine) substrates in series. Oxidative phosphorylation (OXPHOS) capacity for both protocols was induced with the addition of 2 mM ADP, 10 mM glutamate, and 10 mM succinate to the previously mentioned substrates. The uncoupler carbonyl cyanide *p*‐ phenylhydrazone (FCCP) was titrated (1.0 µM per addition up to optimal concentration) to measure electron transport system (ETS) or maximal uncoupled respiration. For all experiments, the integrity of the outer mitochondrial membrane was verified by addition of cytochrome c (Kuznetsov et al., 2004). Samples showing an increase in respiration of more than 15% with the addition of cytochrome c were excluded from final analysis. Data were normalized to wet weight of the tissue.

### 2.11 ^14^C‐labeled ex vivo substrate oxidation

Substrate oxidation studies were performed in homogenized muscle tissue as previously described (Sparks et al., 2013). Briefly, muscle tissue samples were homogenized in buffer containing 250 mM sucrose, 10 mM Tris‐HCl, 1 mM EDTA, and 2 mM ATP (pH 7.4). Homogenates were plated in a modified 48‐well trapping device. Reactions were initiated with the addition of ^14^C‐palmitate and 0.5% fatty‐acid–free BSA and the reaction mixture (124 mM sucrose, 24 mM potassium phosphate monobasic, 200 mM potassium chloride, 2.5 mM magnesium chloride, 2.5 mM L‐carnitine, 0.25 mM malic acid, 20 mM Tris‐HCl, 2.4 mM DTT, 0.25 mM NAD+, 4 mM ATP, and 0.125 mM coenzyme A) and incubated for 2 hr at 37°C. Reactions were terminated by addition of 70% perchloric acid. ^14^CO_2_ was trapped in the adjoining well in 1N NaOH, which was analyzed by a scintillation counter (Tri‐Carb 2810‐TR, Perkin Elmer Inc) to determine CO_2_ production. Remaining muscle homogenates from each well were spun twice for measurement of ^14^C‐labeled acid‐soluble metabolites (ASMs) in 5 ml of Uniscint BD (National Diagnostics). Data were normalized to protein content.

### 2.12 DNA isolation and mitochondrial DNA copy number

Total DNA was isolated from ∼20 mg of skeletal muscle tissue using DNeasy blood and tissue extraction kit (QIAGEN Inc). Quantity and integrity of the DNA was confirmed by a Biotek Synergy 2 platereader (BioTek Instruments, Inc.). All primers and probes were designed using Primer Express version 2.1 (Applied Biosystems, Roche). Relative amounts of mitochondrial DNA (mtDNA) and nuclear DNA were determined by real‐time quantitative PCR as previously described (He et al., 2002).

### 2.13 RNA extraction, real time reverse transcriptase‐quantitative PCR (RT‐qPCR), and microarray

RNA was extracted from ~50 mg of skeletal muscle tissue using the Qiagen RNeasy Fibrous Tissue Mini Kit (Qiagen Inc) (Costford et al., 2010). RNA purity and quantity were determined using a Biotek Synergy 2 plate reader (BioTek Instruments, Inc.). For RT‐qPCR assays, primer‐probe sets were predesigned Single Tube Taqman^®^ Gene expression assays. Reactions were performed using Taqman Fast Virus 1‐step Master reaction mix and the parameters of the Fast Virus 1‐step Standard protocol were followed. (Life Technologies). The ViiA^®^™ 7 Real Time PCR system was used for all reactions (Applied Biosystems, Life Technologies). Gene expression data were normalized to the geometric mean of two internal controls (RPLP0 and GAPDH). Labeling of cRNA and hybridization to Human HT‐12 Expression BeadChips (Illumina) was performed by the Genomics Core at Sanford Burnham Prebys Medical Discovery Institute.

### 2.14 Bioinformatic analysis

The BeadChips array expression data were analyzed using a custom‐built bioinformatic pipeline, including three seamless connected procedures: (a) raw data preprocessing, (b) differentially expressed gene (DEG) analysis, and (c) the assessment of exercise training remodeling effects. The raw data preprocessing follows the protocol established in beadarray, an R/Bioconductor package designed specifically for Illumine array analysis (Dunning, Smith, Ritchie, & Tavare, 2007). Briefly, raw iDAT files from BeadScan were first read, then filtered to remove nonresponding probes. Differences in expression levels across a chip and between chips were then corrected to normalize the signal. Finally, the gene expression values were log_2_‐transformed and quantile normalized for subsequent statistical analysis.

The DEG analyses were performed using Limma, an R/Bioconductor software package for gene discovery. Comparisons were made between the LA and LOS Pre‐exercise groups (baseline differences), as well as LOS Postexercise versus LOS Pre‐exercise (exercise effect). Once DEG sets were identified, functional analysis was performed using clusterProfiler, an R/Bioconductor software package for gene set enrichment analysis (GSEA) (Yu, Wang, Han, & He, 2012). GSEA was performed on KEGG database (Kanehisa & Goto, 2000) to identify significantly enriched biological pathways. Lastly, random forest predictors (Svetnik et al., 2003) was applied with split‐variable randomization to find genes associated with group assignment and rank them according to their classification significances (Liaw & Wiener, 2002). The PCA analysis was performed with all DEGs with *p* < .05 (8586 features). The gene expression data presented in this manuscript has been deposited in the NCBI’s Gene Expression Omnibus and are accessible through the accession number GSE139258 and to reviewers using the following link: https://www.ncbi.nlm.nih.gov/geo/query/acc.cgi?acc=GSE139258 with token gnmdyouqvxgbbit.

### 2.15 Muscle fiber typing

#### 2.15.1 Immunofluorescence and histochemical analysis

Skeletal muscle tissues mounted in OCT blocks were sectioned using a cryotome FSE cryostat (Thermo Scientific Inc). Fiber type was determined by immunohistochemistry performed on 10 μM sections stained using a primary antibody cocktail for 1 hr at room temperature then at 4°C overnight. The antibody cocktail contained the following antibodies: BA‐F8: IgG2b (Type I fibers); 6H1: IgM (Type IIx fibers); SC‐71: IgG1 (Type IIa fibers). All antibodies were diluted 1:50. The secondary antibody cocktail consisted of Alexa Fluor 350 (IgG2b) goat anti‐mouse, Alexa Fluor 555 (IgM) goat anti‐mouse, and Alexa Fluor 488 (IgG1) goat anti‐mouse. All dilutions for secondary antibodies were 1:500. Slides were washed, then stained for wheat germ agglutinin (WGA; Alexa Fluor 647, 50 mg/ml) for 30 min at room temperature in the dark. DAPI staining was used to visualize nuclei (ProLong^®^ Gold Antifade, Life Technologies).

#### 2.15.2 Imaging

Digital images of human skeletal muscle biopsy immunostained sections were acquired using a Nikon Eclipse Ti‐U microscope (Nikon Instruments Inc). Image analysis was performed using NIS‐Elements AR 4.13 software (Nikon Instruments Inc). A control slide was used to determine the exposure used for all subsequent images. Following image acquisition and background correction, image intensity and cross‐sectional area were determined using a macro designed to localize circularity (for boundary detection) and intensity.

#### 2.15.3 Western blot

Skeletal muscle tissue homogenates were prepared in 1x Odyssey Blocking Buffer (LI‐COR, Lincoln). Proteins (15 μg per well) were separated using 10% Mini‐PROTEAN^®^ TGX™ Precast Protein Gel (BioRad). Proteins were then transferred to an Immun‐Blot^®^ Low Fluorescence PVDF Membrane (BioRad) at 100 V for 20 min in 20% methanol. Membranes were blocked in 50% Odyssey Blocking Buffer (LI‐COR, Lincoln, NE)/50% TBS for 1 hr and then incubated in a 1:3,000 dilution of an anti‐Tribbles 1 (TRIB1) antibody (Abgent, San Diego, CA, #AP7726b) or 1:1,000 dilution of Cyclophilin A (Cell Signaling, #2175) antibody in 1x Odyssey Blocking Buffer. Membranes were incubated overnight with shaking at 4°C. Membranes were washed and incubated for 1 hr in TBST Buffer with 1:15,000 IRDye 800CW conjugated goat anti‐rabbit 2° Ab (LI‐COR, 926‐32211). After washing, bands were again visualized and quantified. Levels of TRIB1 were normalized to Cyclophilin A.

### 2.16 Statistical analysis

Statistical analysis was performed using Prism (v6.0, GraphPad Software Inc.) and JMP (v13.2.1). Data are presented as mean ± *SD*, except when indicated differently. Significance was set at *p* < .05. Normality of data was tested using the Shapiro‐Wilk *W*‐test. One‐way ANOVA was used to compare measures across groups followed by Fisher's LSD *post hoc* tests. Pearson correlations were used for normally distributed data. Spearman rank was used for data not normally distributed.

## 3 RESULTS

### 3.1 Participant's characteristics, body composition, and plasma profile

**FIGURE 1:**
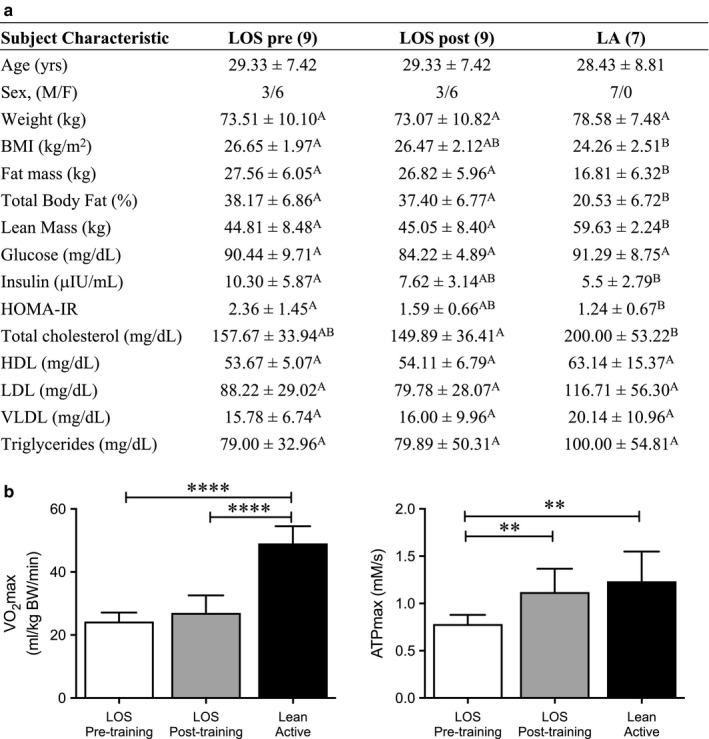
Lean active subjects display higher cardiorespiratory capacity (VO_2_max) and muscle mitochondrial function in vivo (ATPmax) than sedentary subjects. (a) Subject characteristics of the lean/overweight sedentary (LOS) Pre‐ and Postexercise intervention (*n* = 9) and the lean active (LA) (*n* = 7) groups. Letters indicate significance. (b) VO_2_max and ATPmax is shown in the LOS Pre‐ and Postexercise and LA groups. Bars represent mean ± standard deviation. **p < .01 and ****p < .0001 of the indicated comparison by one‐way ANOVA

The participants’ characteristics are shown in Figure 1a. Age was not different across groups, with average age 28.4 ± 8.8 and 29.3 ± 7.4 years for LA and LOS respectively. At baseline (LOS Pre‐exercise compared to LA), body weight did not differ; however, BMI, total fat mass, and fat mass % were significantly lower in the LA group. Likewise, lean mass was significantly higher in the LA group at baseline (LOS Pre‐exercise compared to LA). There were no baseline differences in total cholesterol, LDL, HDL or triglycerides between the LA and LOS groups. Fasting glucose levels were similar between the LOS and LA groups, while insulin levels were lower in the LA group. The LA group was also more insulin sensitive as measured by HOMA‐IR at baseline compared to LOS Pre‐exercise. Aerobic training in the LOS group did not significantly change any of these characteristics.

### 3.2 Cardiorespiratory fitness (VO_2_max) and in vivo mitochondrial function (ATPmax)

VO_2_max and ATPmax were higher in the LA group compared to LOS at baseline (*p* < .0001) (Figure 1b). There was a small but insignificant increase in VO_2_max in the LOS group with aerobic training. However, a large increase in ATPmax to a level nearly comparable to LA was observed in the LOS Postexercise group (Figure 1b). These data demonstrate that the aerobic training protocol produced a robust and significant increase in vivo muscle mitochondrial function in the LOS group.

### 3.3 Effects of exercise on mitochondrial oxidative capacity

**FIGURE 2:**
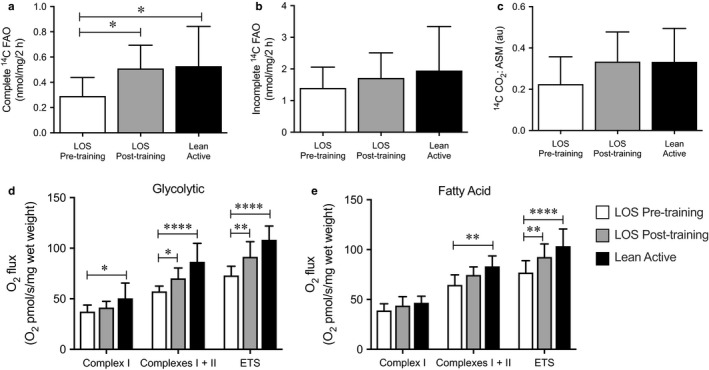
Improvements in ex vivo measures of skeletal muscle fatty acid oxidation and mitochondrial respiratory capacity following the exercise intervention in lean subjects. Complete (a) and incomplete (b) fatty acid oxidation in lean/overweight sedentary (LOS) Pre‐ and Postexercise (*n* = 9) intervention and the lean active (LA) (*n* = 7) groups is shown. (c) The ratio of complete to incomplete fatty acid oxidation is shown as a measure of the efficiency of oxidative metabolism. *p < .05 of the indicated comparison by one‐way ANOVA. Complex I, I + II, and maximal electron transport system (ETS) supported mitochondrial respiration was measured in permeabilized muscle fibers supported by glycolytic (d) and fatty acid (e) substrates in the indicated group. Bars represent mean ± standard deviation. *p < .05, **p < .01, and ****p < .0001 of the indicated comparison by two‐way ANOVA

Exercise training is known to increase muscle oxidative capacity in muscle (Meex et al., 2010; Sparks et al., 2013). Therefore, we measured fatty acid oxidation, as well as mitochondrial respiratory capacity, at baseline and following 18 days of exercise training in these subjects. Baseline complete fatty acid oxidation in muscle was higher in LA compared to the LOS group (Figure 2a). Aerobic training increased complete fatty acid oxidation in muscle of LOS by ~68%, reaching levels comparable levels to the LA group (Figure 2a). Incomplete fatty acid oxidation was not significantly different across groups at baseline or following aerobic training (Figure 2b). In addition, the ratio of complete to incomplete fatty acid oxidation, which reflects the efficiency of oxidative capacity, was comparable Post‐exercise in the LOS group and the LA group (Figure 2c).

High resolution respirometry was also performed on muscle fibers to determine mitochondrial respiratory capacity. These measurements were performed using both glycolytic (glutamate) and fatty acid (palmitoyl‐carnitine) substrates. The LA group had significantly greater baseline complex I, maximal OXPHOS (complex I + II), and maximal uncoupled (ETS) respiratory capacity in muscle supported by glycolytic substrates as compared to LOS (Figure 2d). The LA group also had significantly greater baseline maximal OXPHOS (complex I + II) and maximal uncoupled (ETS) respiratory capacity compared to the LOS group supported by fatty acids (Figure 2e). Aerobic training increased maximal OXPHOS (complex I + II) respiratory capacity in LOS muscle fibers supported by glycolytic substrates (Figure 2d). Uncoupled (ETS) respiratory capacity supported by both glycolytic and fatty acid substrates were also observed with aerobic training in the LOS group (Figure 2d and e respectively).

### 3.4 Muscle fiber type and mtDNA content

**FIGURE 3:**
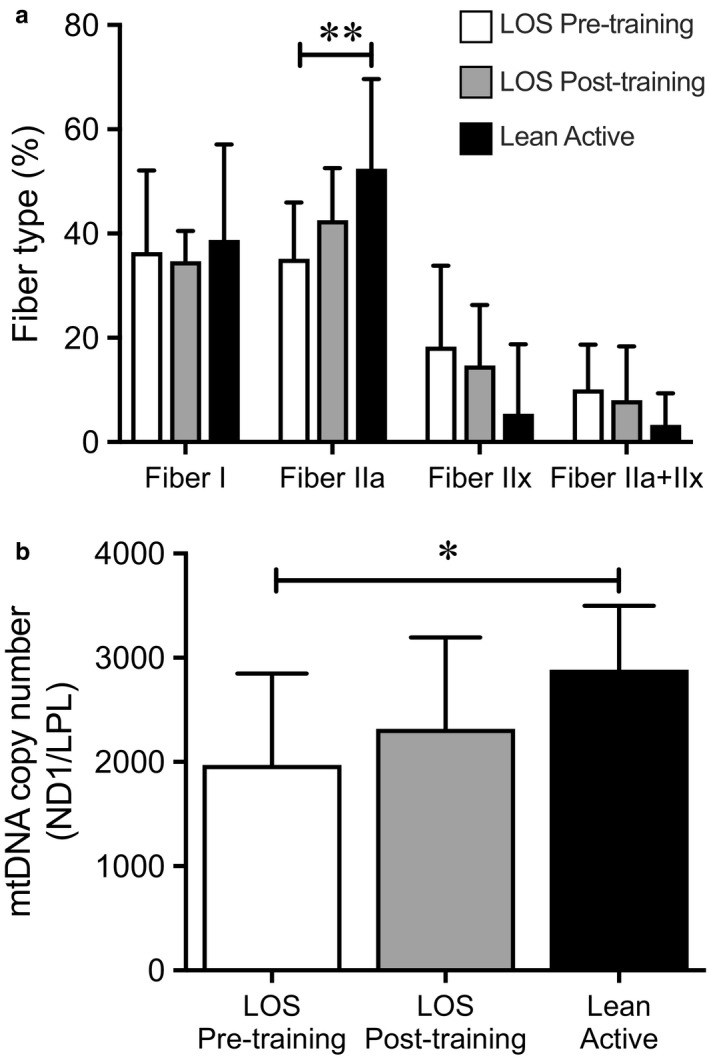
Fiber‐type and mtDNA content did not change during the exercise intervention. (a) The percentage of Type I, IIa, IIx, and hybrid fibers from vastus lateralis biopsies was determined by immunohistochemistry in the lean/overweight sedentary (LOS) Pre‐ and Postexercise (*n* = 9) and lean active (LA) (*n* = 7) groups. (b) mtDNA content was measured in vastus lateralis biopsies in the indicated group. mtDNA is expressed as a ratio of a mitochondrial gene (ND1) to a nuclear control gene (LPL). Bars represent mean ± standard deviation. *p < .05 and **p < .01 of the indicated comparison by one‐way ANOVA

As muscle fiber type is known to greatly influence respiratory capacity and mitochondrial content, muscle fiber type and mtDNA copy number were measured at baseline and following aerobic training. Type IIa fiber type composition was higher in the LA group at baseline (Figure 3a). LA had significantly higher mtDNA content than LOS (Figure 3b). Aerobic training did not change skeletal muscle fiber type composition nor mtDNA copy number (Figure 3a and b respectively).

### 3.5 Exercise training effects on the skeletal muscle transcriptome

**FIGURE 4:**
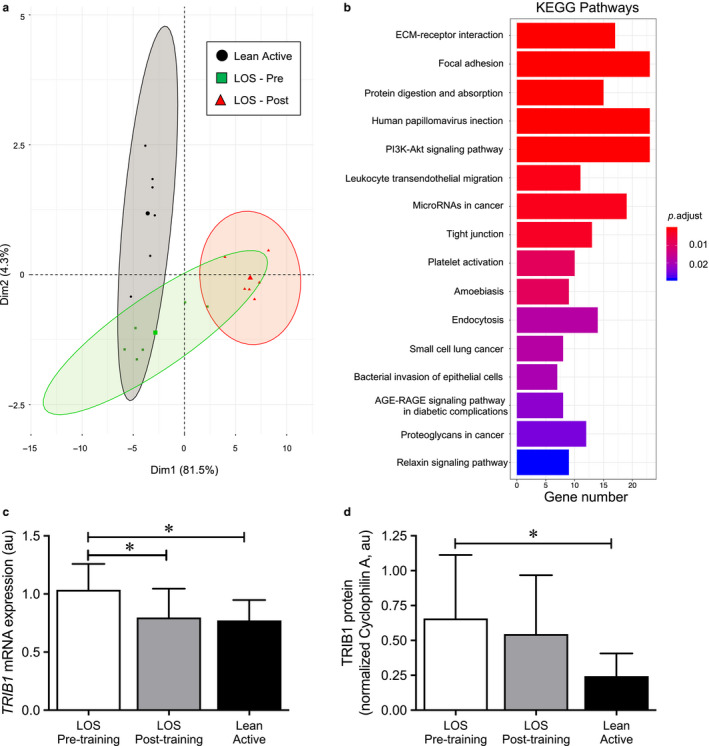
Transcriptome analysis reveals that *TRIB1* is an exercise responsive gene. Microarray analysis was performed on RNA isolated from the lean/overweight sedentary (LOS) Pre‐ and Post‐exercise and lean active (LA) groups (*n* = 6/group). (a) A principal component analysis plot of all differentially expressed genes (DEGs) (*p* < .05) demonstrates clear separation of all three groups. (b) The graph represents significantly enriched KEGG pathways for the DEGs between the LOS Pre‐ and Postexercise groups. The number of DEGs in each pathway is indicated below the graph. (c) Levels of *TRIB1* mRNA were measured by RT‐qPCR in the LOS Pre‐ and Postexercise (*n* = 9) and LA (*n* = 7) groups. (d) TRIB1 protein levels were measured by western blot analysis were also measured in the indicated group. Bars represent mean ± standard deviation. *p < .05 of the indicated comparison by one‐way ANOVA

**FIGURE 5:**
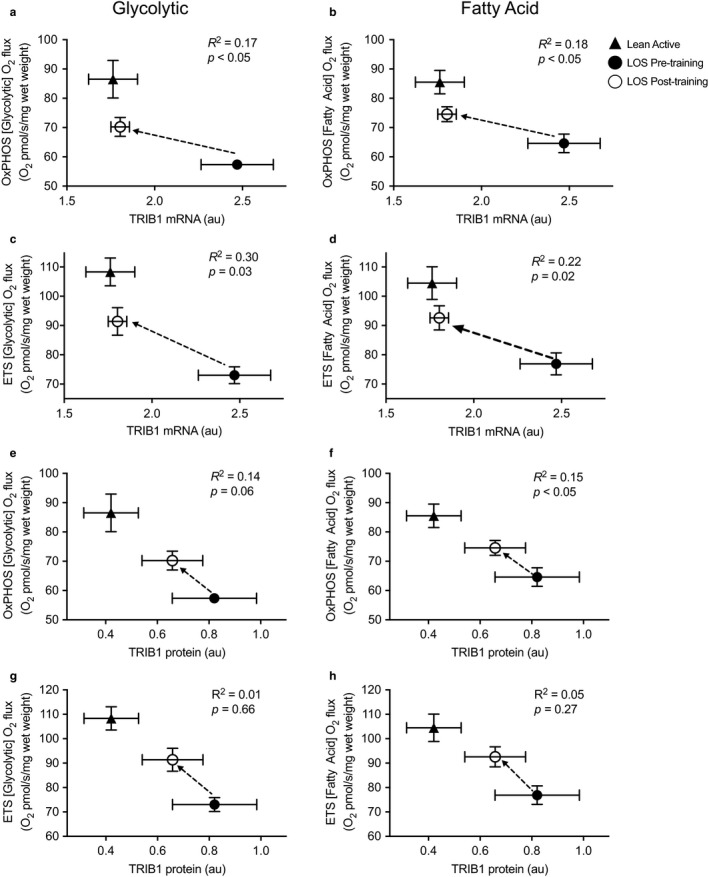
TRIB1 mRNA and protein levels correlate with mitochondrial respiration levels. The association between *TRIB1* mRNA and protein levels with measures of mitochondrial respiration in the lean/overweight (LOS) Pre‐ and Postexercise and lean active (LA) groups were examined. The TRIB1 mRNA and OXPHOS (Complex I + II) or maximal electron transport system (ETS) mitochondrial respiration in permeabilized muscle fibers supported by glycolytic (a and c) or fatty acid (b and d) substrates is shown. The association between TRIB1 protein and OXPHOS and ETS mitochondrial respiration supported by glycolytic (e and g) or fatty acid (f and h) substrates is shown. The dashed arrow depicts the change from LOS Pre‐ to Postexercise associations

To further define the molecular transducers of the aerobic training effects and the inherent distinguishing signature between the LA and LOS groups, microarray analysis was performed on the LA group and Pre‐ and Postexercise in the LOS groups (*n* = 6/group). A principal component analysis (PCA) was performed using all differentially expressed genes (DEGs) of the dataset. The PCA plot shown in Figure 4a demonstrates, as expected, separation between the LA and LOS Pre‐exercise group. Somewhat surprisingly, following the aerobic training, the LOS group was distinctly separated from the Pre‐exercise and LA groups (Figure 4a). Examination of the genes and pathways regulated by aerobic training revealed a large number of genes associated with the extracellular matrix (ECM) including a number of collagen genes (Figure 4b and Figure S1, https://figshare.com/s/eb3145e1716c5783096b). In addition, a number of KEGG pathways identified as being regulated with exercise training were highly connected with multiple shared genes among the pathways (Figure S1, https://figshare.com/s/eb3145e1716c5783096b). The robust activation of ECM and related pathways most likely is a major differentiator of the Postexercise group with the LA and LOS Pre‐exercise group observed in the PCA plot (Figure 4a). We next examined this dataset for genes that were significantly different at baseline between the LOS and LA groups and regulated by aerobic training. Of note, the gene *TRIB1* (Tribbles 1) was expressed at higher levels in the LOS at baseline. Interestingly, the Tribbles family (*TRIB1*, *2*, and *3*) encodes pseudokinases that serve to bind specific substrates of E3 ubiquitin ligases. We confirmed the microarray finding using RT‐qPCR that *TRIB1* expression was significantly lower in the LA group at baseline as compared to LOS (Figure 4c). Interestingly, *TRIB1* expression decreases to a similar level as the LA group following exercise training (Figure 4c). Similar trends were seen with TRIB1 protein, whereby TRIB1 protein levels were significantly higher in LOS Pre‐exercise group compared to LA group and decreased a small but insignificant amount with aerobic training (Figure 4d and Figure S2, https://figshare.com/s/eb3145e1716c5783096b). Additionally, *TRIB1* gene expression showed negative correlations with maximal OXPHOS (complex I + II) and uncoupled (ETS) respiratory capacity in muscle with both glycolytic and fatty acid substrates (Figure 5a–d). Again, although not always significant, TRIB1 protein also trended to be negatively correlated with maximal OXPHOS and ETS respiratory capacity with glycolytic and fatty acid substrates (Figure 5e–h). These findings support the notion that TRIB1 may be associated with the trained status and possibly mitochondrial respiratory capacity in skeletal muscle.

## 4 DISCUSSION

Sedentary and/or overweight individuals are characterized by impaired skeletal muscle mitochondrial oxidative metabolism when compared to more active people (Bajpeyi et al., 2011; Phielix et al., 2012; Sparks et al., 2005, 2013). Exercise training can improve muscle mitochondrial capacity in these individuals (Meex et al., 2010). This difference in muscle mitochondrial capacity is influenced by underlying molecular mechanisms. For example, lower expression of PGC‐1α—a key regulator of mitochondrial biogenesis and oxidation—has been associated with lower muscle mitochondrial capacity in these individuals (Mootha et al., 2003; Patti et al., 2003). PGC‐1α expression is markedly induced by a single bout of exercise (Pilegaard, Saltin, & Neufer, 2003). In addition, an increase in PGC‐1α gene expression seems to be important for exercise training‐induced improvements in muscle mitochondrial capacity (Fernandez‐Marcos & Auwerx, 2011; Lira et al., 2010). Some studies, however, have found that improvements in muscle mitochondrial capacity can occur before PGC‐1α gene expression is elevated (Wright et al., 2007). As such, additional molecular regulators that might play a role in the early adaptations of muscle mitochondria to exercise need to be identified. Our study confirms that muscle mitochondrial capacity is impaired in overweight and sedentary people when compared to active people. In addition, our results show that an exercise program of 18 days increases muscle mitochondrial capacity in lean/overweight sedentary individuals to levels observed in active individuals.

Muscle mitochondrial capacity—measured in vivo and ex vivo—and complete fatty acid oxidation rates were higher in active people compared to overweight and/or sedentary people. These differences at baseline were paralleled by higher mitochondrial DNA copy number in active individuals. Differences in muscle mitochondrial capacity can originate from differences in both mitochondrial content and intrinsic capacity of the mitochondria. In elderly subjects, lower muscle mitochondrial capacity in sedentary individuals compared to active individuals is likely due to lower amounts of mitochondria (Distefano et al., 2018). In patients with type 2 diabetes, lower muscle mitochondrial capacity was reported to be independent of mitochondrial content, and thus more related to lower intrinsic capacity of the mitochondria (Phielix et al., 2008). Interestingly, 12 weeks of aerobic and resistance exercise training improved muscle mitochondrial capacity in patients with type 2 diabetes and in obese healthy individuals by increasing mitochondrial content in the muscle (Phielix, Meex, Moonen‐Kornips, Hesselink, & Schrauwen, 2010). We here observed that lean/overweight sedentary individuals improved muscle mitochondrial capacity following an exercise program of 18 days, with some indices for mitochondrial capacity (ATPmax) reaching levels that were observed in the active individuals. However, it is important to note that the LA group were physically active but not necessarily highly trained athletes. Muscle mitochondrial content or fiber type composition did not change (Figure 3), suggesting that improvements in muscle mitochondrial capacity occurred due to intrinsic changes of the mitochondria. While it is possible that other measurements of mitochondrial content such as cardiolipin levels could be increased, these data does suggest that short‐term exercise training can increase mitochondrial capacity even in the absence of a change in content.

These improvements in muscle mitochondrial capacity, however, were not completely reflected in improvements in VO_2_max, which might suggest that adaptations of muscle mitochondrial capacity and cardiorespiratory fitness to exercise occur somewhat independently. In our study, we observed a small but insignificant increase in VO_2_max. Indeed, and not unexpectedly, not all participants displayed an increase in cardiorespiratory fitness. This may be related to the amount and overall duration of the training program as others have reported a dose–responsiveness of VO_2_max increases to the amount and length of training (Montero & Lundby, 2017). It is also possible that the mitochondrial adaptations occur prior to changes in VO_2_max that are not evident in this relatively short intervention. A number of studies have reported parallel improvements in mitochondrial capacity and cardiorespiratory fitness (Meex et al., 2010; Phielix et al., 2010); however, some have not found this direct relationship (Pesta et al., 2011; Stephens et al., 2018), and discrepancies may be due to deviations in exercise intensity, duration, or mode (Huang et al., 2016; Seals, Hagberg, Hurley, Ehsani, & Holloszy, 1984).

To gain a better understanding of the molecular mechanisms and pathways underlying differences in skeletal muscle mitochondrial capacity between the LOS and LA groups, as well as the effects of aerobic training, we performed microarray analysis of RNA isolated from muscle biopsies at baseline and following training. Interestingly, we did not observe a large number of nuclear‐encoded mitochondrial genes regulated by the exercise intervention. This may be due to the timing of the last exercise bout with the muscle biopsy. However, this is also consistent with our measurement of mtDNA copy number that was not significantly regulated during the exercise training. Genes that were regulated by aerobic training and that were different at baseline between the LOS and LA groups were of particular interest. We found that Tribbles pseudokinase 1 (*TRIB1*) was significantly lower in the LA group and was decreased with exercise (Figure 4c). *TRIB1* is one of three mammalian tribbles genes (*TRIB1‐3*) that encode scaffold or adaptor proteins to facilitate degradation of target proteins via the ubiquitin proteasome system (Lohan & Keeshan, 2013). The Tribbles pseudokinases have been increasingly recognized as a major regulator of multiple cellular and physiological processes (Bauer, Yenilmez, & Rader, 2015). *TRIB1* has been previously identified as a regulator of inflammation in mouse adipose tissue (Ostertag et al., 2010). In humans, *TRIB1* in liver regulates hepatic lipid metabolism and is associated with increased liver fat content and cardiovascular disease (Bauer et al., 2015). Our study, however, is the first to describe a potential role for *TRIB1* in skeletal muscle. Interestingly, *TRIB3* has been shown to be higher in the skeletal muscle of insulin resistant individuals compared to insulin sensitive individuals, and has been directly associated with whole‐body insulin resistance and high fasting plasma glucose (Liu et al., 2010). Knockdown of *TRIB3* in skeletal muscle improved insulin sensitivity in a rat model for insulin resistance (Weismann et al., 2011); however, rats exposed to short‐term exercise training improved glucose tolerance without changes in skeletal muscle TRIB3 protein (Canciglieri et al., 2018). TRIB3 has also been implicated in the regulation of muscle mass, as well as exercise capacity (An et al., 2014; Choi et al., 2019); however, RNA‐sequencing data we have collected from human *vastus lateralis* muscle demonstrates that *TRIB1* is the predominant form expressed in human muscle with minimal expression of *TRIB3* (data not shown). Therefore, these observations indicate that alterations in *TRIB1* expression may be involved in the adaptation to exercise in human skeletal muscle. Given its known function as a scaffold protein to facilitate protein degradation, TRIB1 may be directly influencing the stability or levels of key mitochondrial proteins. However, future studies are needed to assess the precise molecular function for TRIB1 in muscle and its role in mediating the response to exercise.

In conclusion, we find differences in muscle mitochondrial capacity between active and sedentary people. Additionally, even a relatively short exercise training regimen can improve muscle mitochondrial capacity and increase muscle oxidative capacity, by some measures, to levels observed in active individuals. These changes were not associated with changes in fiber‐type or mitochondrial content suggesting intrinsic improvements in muscle mitochondrial capacity. Furthermore, microarray profiling identified unique molecular signatures of aerobic training in the sedentary group. Using this approach, we discovered changes in *TRIB1* expression that correlated with various measures of mitochondrial capacity in skeletal muscle. Our data therefore point toward *TRIB1* as a potential regulator of the effects of aerobic training in human skeletal muscle.

## CONFLICT OF INTEREST

This research was funded through a partnership with Takeda Pharmaceuticals. SRS and SP received research support from Takeda Pharmaceuticals, Inc. The other authors have no conflicts of interest. None of the funding sources played a role in the collection, analysis, or interpretation of the data or in the decision to submit the manuscript for publication.

## AUTHOR CONTRIBUTIONS

SS and LS conceived the study concept and design. RV, BB, LS, SP, MP, FY, GY, and NS wrote the manuscript, elaborated tables and figures, and/or participated in the analysis and interpretation of data. FY and AH participated in data analysis. SS and RP critically reviewed the manuscript. LS is the guarantor of this work and as such had full access to all the data in the study and takes responsibility for the integrity of the data and the accuracy of the data analysis. All authors read and approved the final manuscript.
