# Supplementary material for: An improvement in skeletal muscle mitochondrial capacity with short‐term aerobic training is associated with changes in Tribbles 1 expression
Source: Physiol Rep. 2020 Jun 19;8(12):e14416. doi: 10.14814/phy2.14416 (PMC7305239; doi:10.14814/phy2.14416)
Supplement: Supplementary file 1 [file PHY2-8-e14416-s001.pdf]

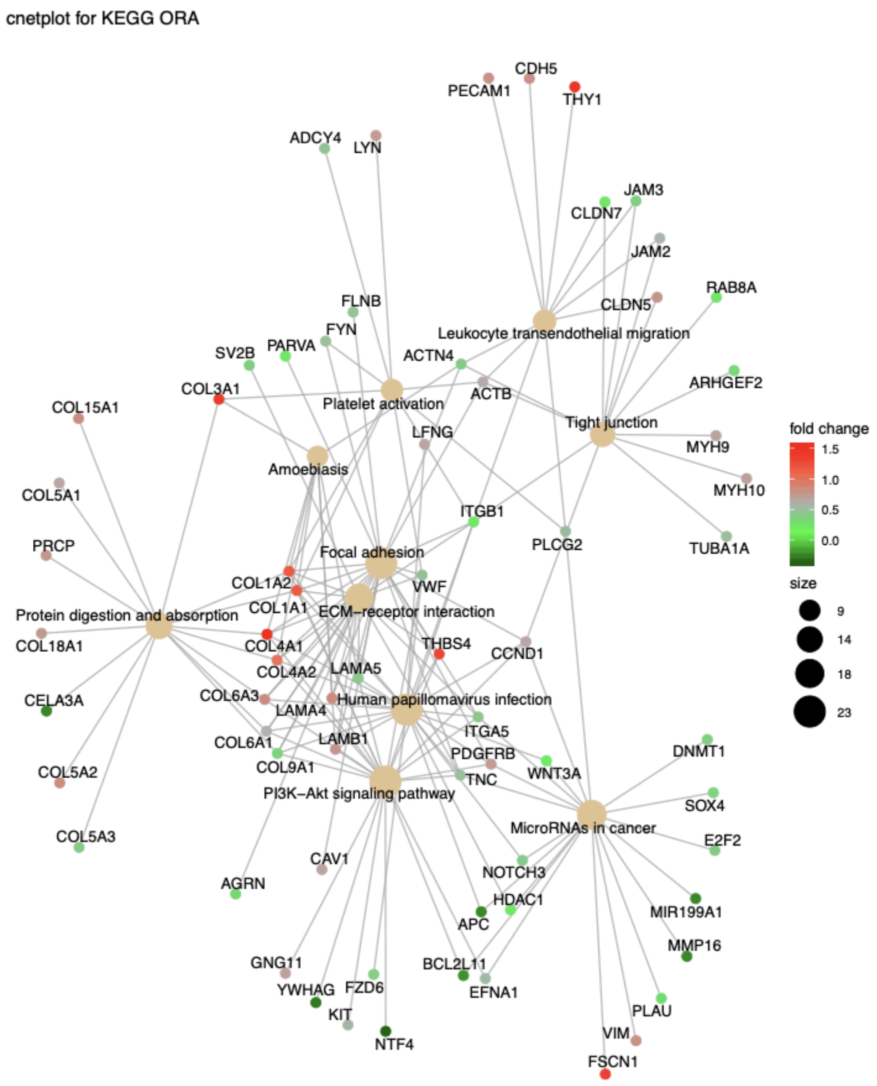

Supplemental Figure 1. A high degree of connectivity between KEGG pathways regulated by the exercise intervention. The R/Bioconductor software, clusterProfiler, was used to identify the interconnectivity of the significantly enriched KEGG pathways in the comparison between the lean/overweight sedentary (LOS) Pre- and Post-training groups. The heat map shows the $\log_2$ fold-change of expression of each gene. The size of the brown circles indicating each KEGG pathway represents the number of DEGs in each pathway.
