# Supplementary material for: An improvement in skeletal muscle mitochondrial capacity with short‐term aerobic training is associated with changes in Tribbles 1 expression
Source: Physiol Rep. 2020 Jun 19;8(12):e14416. doi: 10.14814/phy2.14416 (PMC7305239; doi:10.14814/phy2.14416)
Supplement: Supplementary file 2 [file PHY2-8-e14416-s002.pdf]

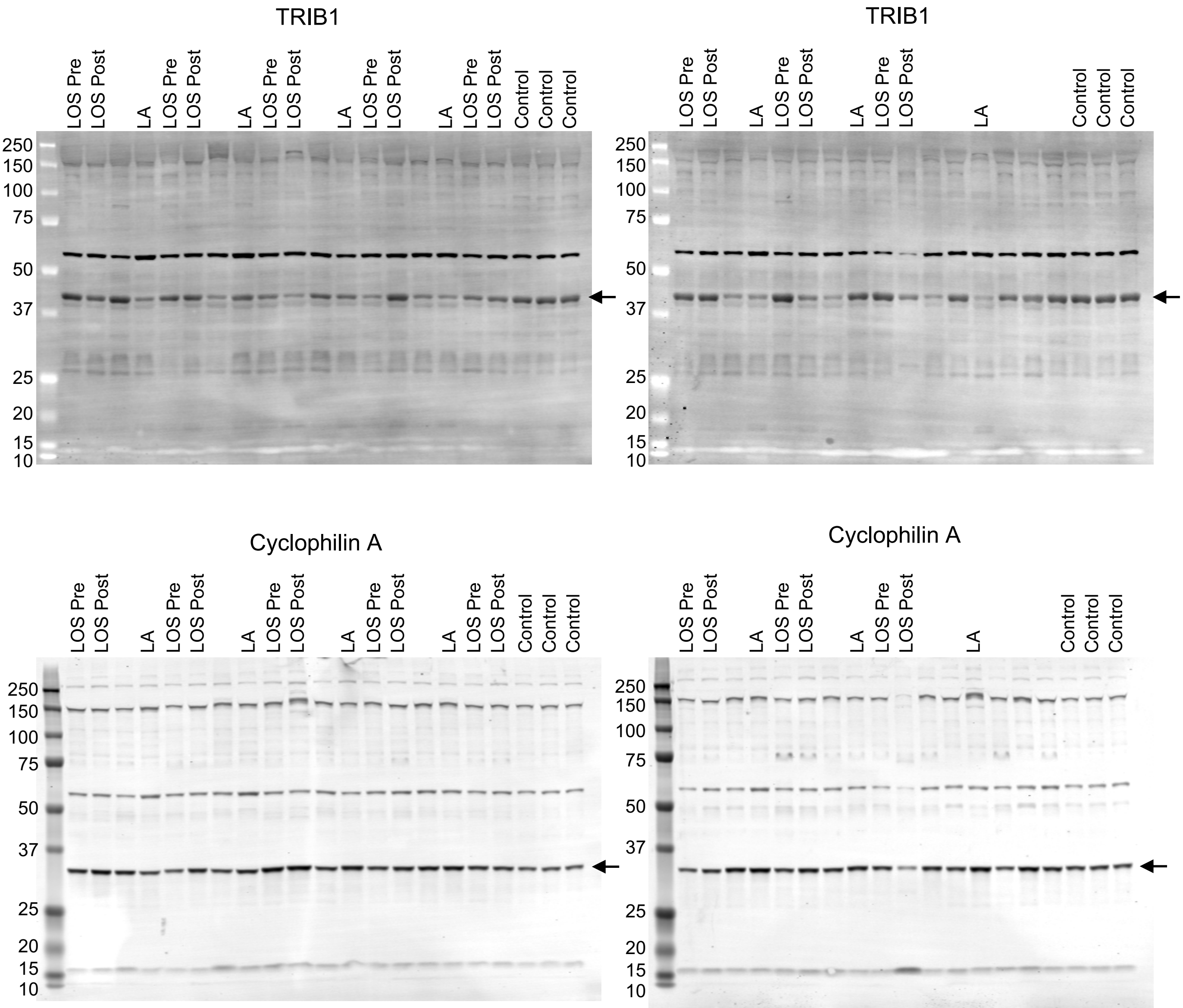

Supplemental Figure 2. Western blot analysis TRIB1 protein. The full uncropped gels for the TRIB1 and Cyclophilin A westerns are shown. The group for each subject is indicated above the lane. Unmarked lanes are samples that are from a group that was not included in the manuscript. Control lanes represent pooled muscle lysates and were included as a loading control across the different gels. An arrow indicates the band for TRIB1 or Cyclophilin A protein.
